# Anxiety, fear, panic: An approach to assessing the defensive behavior system across the predatory imminence continuum

**DOI:** 10.3758/s13420-021-00509-x

**Published:** 2022-02-02

**Authors:** Ann N. Hoffman, Jeremy M. Trott, Anna Makridis, Michael S. Fanselow

**Affiliations:** 1 Department Psychology, University of California Los Angeles, Los Angeles, CA, USA; 2 Staglin Center for Brain and Behavioral Health, Los Angeles, CA, USA; 3 Department of Neurosurgery, David Geffen School of Medicine, University of California Los Angeles, Los Angeles, CA, USA; 4 Department of Psychiatry and Biobehavioral Sciences, University of California Los Angeles, Los Angeles, CA, USA

**Keywords:** Associative learning, Behavior systems, Fear conditioning, Nonassociative, Defensive behavior, Predatory imminence continuum

## Abstract

In order to effectively thwart predation, antipredator defensive behaviors must be matched to the current spatio-temporal relationship to the predator. We have proposed a model where different defensive responses are organized along a predatory imminence continuum (PIC). The PIC is a behavior system organized as a sequence of innately programmed behavioral modes, each representing a different interaction with the predator or threat. Ranging from low threat to predator contact, the PIC categorizes defense modes as pre-encounter, post-encounter, and circa-strike, corresponding to states of anxiety, fear, and panic, respectively. This experiment examined if the same significant stressor caused overexpression of all defensive responses along the PIC, including anxiety-like behavior, freezing, and panic-like responses. Female and male mice were exposed to acute stress that consisted of a series of ten pseudorandomly presented unsignaled footshocks (or no shocks). Mice were subsequently tested on a battery of tasks to assess stress effects on pre-encounter (anxiety-like), post-encounter (fear), and circa-strike (panic-like) behaviors. Results revealed that following stress, mice exhibited increased anxiety-like behavior shown through reduced average velocity within a modified open field. Furthermore, stressed mice showed increased fear following a single footshock in a new context as well as an increase in reactivity to white noise in the original stress context, with stressed mice exhibiting a more robust circa-strike-like response than controls. Therefore, significant stress exposure influenced the defensive states of anxiety, fear, and panic across the predatory imminence continuum. This research could therefore reveal how such responses become maladaptive following traumatic stress in humans.

## Introduction

In a behavior systems approach, researchers take advantage of the strengths of laboratory learning models with ethological observations to define the organization of behavior that reflects adaptive benefits to the animal (Domjan & Gutierrez, 2019; Timberlake, 1994). Well-studied behavior systems include both appetitive (food procurement and sexual behavior) and aversive (defense) systems, which have opposing basic rules in moving between system modes. In the defensive behavior system, under conditions of threat such as predation, an animal’s behavioral response becomes limited to adaptive species specific defense reactions (SSDRs; Bolles, 1970). An influential model of SSDR selection well conserved across species is the Predatory Imminence Continuum (PIC) theory (Fanselow & Lester, 1988; Perusini & Fanselow, 2015). The PIC states that qualitatively distinct defensive behaviors are matched to the spatial, temporal, and psychological distance from physical contact with a life-threatening situation. Each defense mode across the PIC has a unique antecedent (causal) condition that engages a distinct set of behaviors. Increased risk of threat leads to modifications in behavior in an effort to reduce the likelihood of predation. For example, rodents freeze when they detect a predator but show a vigorous burst of activity to contact by the predator. The three defense modes, pre-encounter, post-encounter, and circa-strike, map well onto states of anxiety, fear, and panic in both behavior and neural circuits in mammals (Mobbs et al., 2009; Perusini & Fanselow, 2015).

Appropriate deployment of the Predatory Imminence Continuum allows animals to adaptively match defensive behavior to a particular threat, while protecting non-defensive behavior to allow for foraging activities and other appetitively motivated activities. However, prior experience with significant threat or stressor exposure can alter an animal’s future adaptation to threat and therefore shift their SSDR pattern to increased defense-compromising behaviors satisfying other needs. This is the basis of stress and trauma-related disorders such as post-traumatic stress disorder (PTSD). In some, significant trauma exposure can lead to elevated defense states such as chronic hyperarousal and exaggerated reactivity to minor stressors and cues associated with or generalized to the trauma (American Psychiatric Association, 2013). Animal models of stress-based fear learning include the stress-enhanced fear learning (SEFL) protocol as a model of PTSD (Perusini et al., 2016; Rau et al., 2005). In this design, rodents in the stress condition are exposed to a series of unpredictable, unsignaled footshocks (1 s/1 mA; 15 shocks for rats over 90 min, 10 shocks for mice over 60 min), whereas controls are left undisturbed for the same amount of time (Rajbhandari et al., 2018). Animals in both conditions are later introduced to a novel context and receive a single footshock, before returning to the home cage. When tested for post-encounter defense (freezing behavior) in the single-shock context, animals that have received the prior stress exhibit robust and reliable exaggerated fear compared to the non-stressed control group. Across various experiments using the SEFL model, the initial significant stressor exposure has effectively led to increased defensive phenotypes including enhanced future fear learning (Perusini et al., 2016; Poulos et al., 2015; Rajbhandari et al., 2018; Rau et al., 2005), increased anxiety-like behavior in classical tasks such as open field and elevated plus maze (Perusini et al., 2016), exaggerated startle response (Perusini et al., 2016), and even has modeled relevant comorbidities such as increased voluntary alcohol consumption (Meyer et al., 2013). These separate studies collectively show that a single significant stress event alters an animal’s defensive state to enhanced and exaggerated SSDRs across species, sexes, and the lifespan. However, most research probing significant stress effects on defensive state including pre-encounter, post-encounter, and circa-strike-related behaviors take a between-state approach to capture its effects on a single element in the continuum.

The present experiment sought to examine if exposure to a prior stress event leads to overexpression of all defensive responses along the PIC within-subject, including anxiety-like behavior, freezing, and panic-like responses. Consistent with the SEFL model used in our lab (Rajbhandari et al., 2018), following exposure to significant acute stress (or no stress), mice were tested on a battery of tasks to assess stress effects on pre-encounter (anxiety-like), post-encounter (fear), and circa-strike (panic-like) behaviors, where testing for each defense state occurred in order of increasing predatory imminence (i.e., from potential danger to close contact). This design provides a full picture of how the stress manipulation affects defensive shifts across the PIC and addresses a gap in the literature by linking shifts in defensive responding to prior experience. Additionally, this approach adds to the field with broad translational relevance for future mechanistic investigation of anxiety and stress-related disorders and reduces the need for common between-state designs that investigate anxiety-like, fear-like, and panic-like behaviors separately.

## Methods

### Subjects

Adult female and male C57BL/6J mice (Jackson Labs; *n* = 16, eight females, eight males) were individually housed and maintained on a 12-h light/dark cycle with food and water ad libitum. All experiments were performed during the light phase of the light cycle. All animals were handled ~1 min/day for 4 days prior to the start of the experiments. The experiment was conducted with approval from the University of California Los Angeles Institutional Care and Use Committee (protocol #09–107).

### Apparatus

**Fig. 1:**
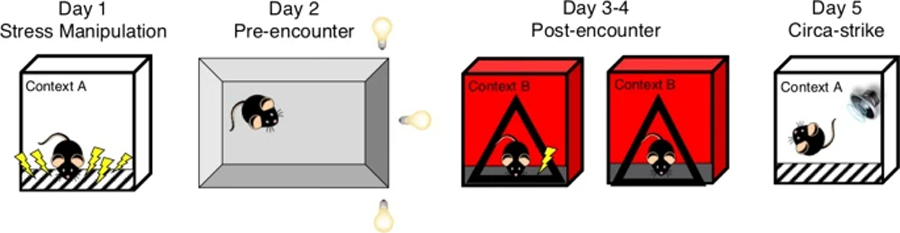
Experimental design. Day 1, Stress manipulation: 10 or 0 unsignaled footshocks over 60 min in Context A. Day 2, Pre-encounter defense: Light gradient open field. Days 3–4, Post-encounter defense: Single-shock fear conditioning and test in Context B. Day 5, Circa-strike defense: Reactivity to white noise in Context A

Behavioral testing was conducted in MedAssociates fear-conditioning chambers (30.5 × 24.2 × 21cm), controlled by compatible VideoFreeze software (MedAssociates, St. Albans, VT, USA). Contexts A and B differed on several features including configuration of the chamber, physical room location, transport method, grid floors, lighting condition, and odor. The experimental design is outlined in Fig. 1.

### Stress manipulation

Mice were subjected to our significant stress protocol adapted for mice based on our well-established stress-enhanced fear learning model of fear sensitization (Perusini et al., 2016; Rajbhandari et al., 2018; Rau et al., 2005). On Day 1, mice were placed in a novel context chamber (Context A) and after a 180-s baseline period were presented with ten pseudorandom presentations of unsignaled 1 s/1.0 mA footshocks over 1 h; no stress controls were placed in the chamber for equivalent duration without shocks.

### Pre-encounter defense (anxiety)

Anxiety-like behavior was assessed on Day 2 using the light gradient open field task (Godsil et al., 2005; Godsil & Fanselow, 2004). Classical anxiety-like behaviors were measured (locomotion, velocity, thigmotaxis), with the addition of the sudden onset of a bright light at one end of the rectangular arena that causes an activity response to the change in environmental stimuli. The apparatus consisted of a clear plastic rectangular open field (69 cm long × 34 cm wide × 30 cm high) placed in the center of a small testing room. A lamp was positioned outside each end of the arena, facing down so as to not directly illuminate the inside of the arena. LED bulbs were used to maintain temperature during the light-on condition on the lit side of the arena. An overhead camera recorded animal behavior throughout the task onto a computer outside the testing room. Video was analyzed using Ethovision software (Noldus; Leesburg, VA). The open field was divided into four zones, where during the light-on phase of the task, zone 1 was the brightest and closest to the lamp, zone 4 was the darkest on the distal end from the lamps, and zones 2 and 3 were of descending illumination along the gradient. The 12-min task was divided in three phases. The mouse was placed in the center of the arena and allowed to explore in the dark for the first 4 min. Then, the lamps illuminated one side of the rectangular arena, creating a gradient across the four zones. Mice explored during the light-on phase for 4 min before the light turned off and the animal explored for the remaining 4 min. Average velocity and time spent in zones were analyzed across phases of the 12-min task. The light-on side was counterbalanced across trials and conditions to eliminate any bias or side preference. Anxiety-like behavior was measured by average velocity and time spent in zones closest to and farthest from the light during the light-on phase. Reduced velocity and more time spent farthest from the light are interpreted as more pre-encounter/anxiety-like behavior.

### Post-encounter defense (fear)

On Days 3–4, post-encounter defense was assessed and followed our typical stress-enhanced fear learning procedure. All groups were subjected to single-shock contextual fear conditioning in a novel context (Context B) distinct from the stress context (Context A). Mice were transported to and placed in Context B and after a 180-s baseline period, received a single 2 s/1 mA footshock, and removed from the chamber 30 s later. Consistent with our SEFL protocol in mice, we increase the duration of the single shock to 2 s to reduce floor levels of fear conditioning for the no stress groups (Rajbhandari et al., 2018). The next day, mice were transported back to Context B and percent time freezing was measured across an 8-min test. Freezing behavior was scored using the VideoFreeze automated software (MedAssociates, St. Albans, VT, USA). In this program, adjacent frames are compared to assess amount of pixel change across frames to produce an activity score. Freezing is scored by a set threshold level manually calibrated to a highly trained observer (MSF).

### Circa-strike defense (panic)

The rodent circa-strike activity burst is characterized by sudden and rapid flight and/or jumps in attempt to escape contact with a predator (Fanselow & Lester, 1988). These behaviors are readily observed in an already frightened rodent when there is a sudden change in stimulus condition (Trott et al., submitted). Circa-strike defensive behavior was assessed on Day 5 as reactivity to 75 dB white noise in the stress context. We intentionally tested the mice in the same stress context (Context A) rather than a neutral context in order to assess a shift between pre-encounter (cautious exploration) and post-encounter defense (freezing) for the no-stress group, compared to the shift between post-encounter (contextual freezing in the stress context) to circa-strike (activity burst) upon noise presentations. Mice were transported back to the stress context (Context A) and after 180 s were presented with 16 trials of 10 s/75 dB white noise. Percent time freezing during the baseline period as well as during the 10-s intervals preceding each noise trial was measured.

Reactivity to noise was measured in three ways: peak activity ratio (PAR), darting, and a velocity map. These measures for circa-strike have been validated in other studies from our lab and show strong support for nonassociative, stimulus-elicited, flight-like circa-strike behavior (PAR, darting, velocity analysis: Trott et al., submitted; PAR: Fanselow et al., 2019). Changes in the magnitude of response to noise and group differences between no stress and stress conditions across metrics described below were interpreted as effects of prior stress.

### Peak activity ratio (PAR)

We measured bursts of activity as a ratio of the animal’s peak activity during a noise trial relative to its peak activity during the same interval just prior to the given trial. PAR was calculated using the raw Videofreeze (MedAssociates) activity score, which compares the amount of change in pixels between adjacent video frames collected at 30 frames/s. We took the maximum activity score (i.e., the greatest degree of pixel change) during a designated interval (10-s noise trial or pre-noise interval). PAR was calculated as the maximum activity score (During Noise / (During Noise +Pre Noise)), where During Noise = 10 s Noise and Pre Noise = 10 s before that Noise trial. For this measure, a value of 0.5 indicates there was no change in the peak activity from before and during the noise trial. A PAR approaching 1.0 indicates a vigorous burst of activity during the noise trial that far exceeded its baseline.

### Darting

We also measured darting behavior as adapted from Gruene et al. (2015). VideoFreeze video files were analyzed with EthoVision XT (Noldus; Leesburg, VA, USA) to determine animal velocity across testing sessions in response to stimulus presentation using a center-point tracking with a velocity sampling rate of 3.75 Hz. Velocity data were exported, organized, and imported to R (R Core Team) and darts were detected using a custom R code with a minimum velocity of 22.9 cm/s and a minimum interpeak interval of 0.8 s, with thresholds based on the 99.5th percentile baseline velocity data from several prior experiments, and validated with manual scoring of darts. Number of darts per noise trial were transformed to be represented and analyzed as dart rate (darts/min). PAR reflects the greatest amplitude activity response, while darts reflect the frequency of large movements.

### Velocity map

Additionally, we performed a microanalysis of the magnitude of noise reactivity during the circa-strike test by binning velocity data into .533-s bins. Binned velocity data were averaged across the early session (first four trials) as well as the whole session (16 trials) surrounding each trial (pre-stimulus period, Noise stimulus, post-stimulus) to determine the temporal pattern of circa-strike reactivity within and across white-noise presentations.

### Data analysis

Behavioral data were analyzed in SPSS as a mixed-factors ANOVA for stress condition and sex across time or trial. When significant interactions were detected at *p* < 0.05, contrasts for simple main effects were performed at each timepoint.

## Results

### Stress manipulation

Percent time freezing during each pre-shock interval (10 s prior to each shock trial) was analyzed by a mixed-factors ANOVA for stress condition and sex across trials. A significant stress x trial interaction was detected (F(9,108) = 16.673, *p* < 0.001). Stressed mice froze significantly more than the no-stress group across trials 2–10, following the first footshock (trial 2, *p* < 0.05; trials 3–10, *p* < 0.001). Levels of freezing reached asymptotic levels for the stress group (86–99% freezing trials 6–10) whereas the no-stress group showed little to no freezing (6–13% trials 6–10), data not shown. It should be noted that recorded levels of freezing in the latter portion of the 1-h stress session for the no-stress group are likely a reflection of sleeping or other inactivity rather than defensive freezing behavior. No other effects were statistically significant.

### Pre-encounter defense (Anxiety)

**Fig. 2:**
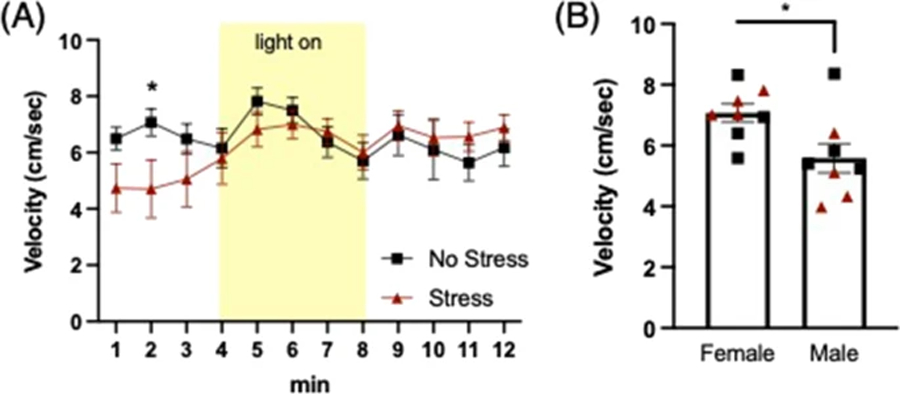
Pre-encounter defense: Light gradient open field. **a** A stress x time interaction revealed that prior stress decreased exploration velocity in the initial dark phase (*p = 0.015). **b** A main effect of sex revealed that female mice had higher velocity on average vs. males (*p = 0.018), regardless of stress history (black squares = No stress; red triangles = Stress)

Average velocity (cm/s) in the open field was analyzed in 1-min bins across the 12-min task. A mixed-factors ANOVA revealed a significant time x group effect (F(11,132) = 2.318, *p* = 0.012), and a main effect of sex (F(1,12) = 7.436, *p* = 0.018). Post hoc comparisons for simple main effects showed that stressed mice had reduced velocity during min 2 (t(14) = 2.091, *p* = 0.05), compared to the non-stressed group, regardless of sex (Fig. 2a). The main effect of sex showed that overall, females had higher velocity than males, regardless of stress condition (Fig. 2b). No significant effects were observed for time spent in zones across dark/light phases of task.

### Post-encounter defense (Fear)

All mice received single-shock contextual fear conditioning in Context B, a novel context relative to the stress context. No differences in freezing were detected during the baseline period prior to the footshock, indicating no contextual fear generalization to context B. Shock reactivity was analyzed by a two-way ANOVA for stress condition and sex as measured by the maximum motion index (largest activity score during shock) for the single 2-s footshock in context B, and revealed a significant main effect of stress (F(1,12) = 21.346, *p* = 0.001), where stressed mice showed reduced reactivity to the shock relative to the no-stress group.

**Fig. 3:**
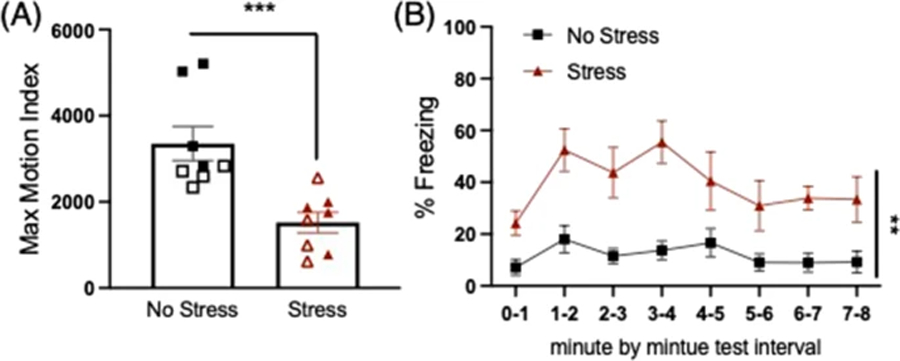
Post-encounter defense: single shock fear conditioning. **a** The stressed group showed reduced shock reactivity to the single shock in context B (***p = 0.001). Open symbols represent females, closed symbols represent males although there were no significant sex effects or interactions. **b** Prior stress increased freezing during fear memory test the following day in context B; (**p = 0.003, main effect Stress vs. No stress)

The next day, mice were returned to Context B and tested for freezing to the context associated with the single shock across an 8 min test. When analyzed across 1 min time bins, a mixed-factors ANOVA revealed a significant effect of time (F(7,84) = 4.448, *p* < 0.001), where freezing levels dynamically changed across the 8 min (see Fig. 3b). There was also a significant main effect of stress condition, where the stressed group showed significantly higher levels of freezing during the test compared to the no-stress group (F(1,12) = 14.465, *p* = 0.003).

### Circa-strike defense (Panic)

**Fig. 4:**
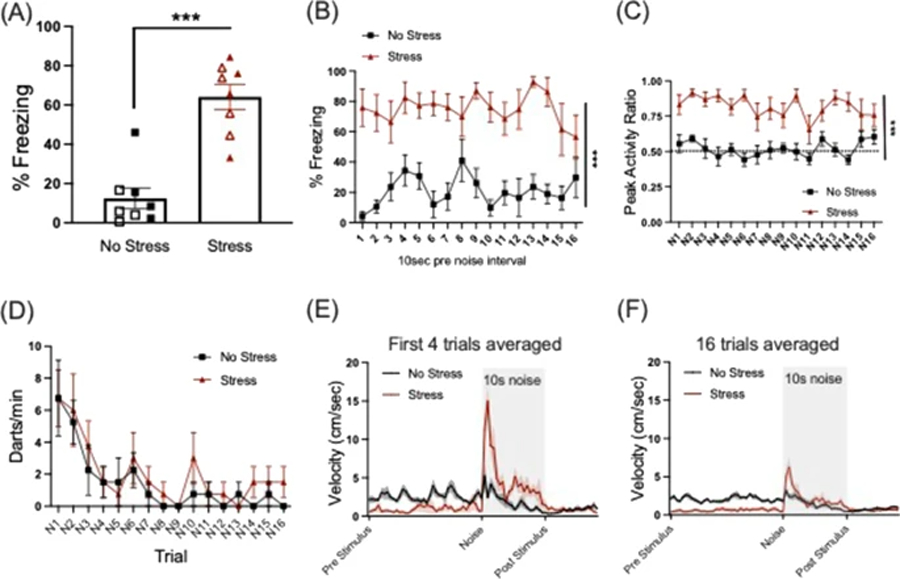
Circa-strike defense: Reactivity to white noise in stress context. **a** The stressed group showed increased baseline freezing during the first three minutes in Context A (***p < 0.001). Open symbols represent females, closed symbols represent males although there were no significant sex effects or interactions. **b** Stress group showed robust freezing in Context A between trials of 75 dB white noise (***p < 0.001 Stress vs. No Stress). **c** Prior stress increased reactivity to trials of 75 dB white noise as measured by peak activity ratio. The no-stress group showed little reactivity to white noise. (***p < 0.001 Stress vs. No Stress.) **d** No differences between groups for dart rate across noise trials. **e** Micro bins (0.533 s) velocity traces for pre-stimulus, noise, and post-stimulus period averaged for the first four trials. Prior stress reduced velocity during pre-stimulus period and robustly increased peak velocity at onset of noise period. **f** Velocity traces averaged for all 16 trials of session. **e, f** Note similar patterns for groups across stimulus periods and differences in magnitude between early session (**e**) and whole session (**f**). The shaded area represents the 10-s noise period

On day 5, all mice were returned to Context A and received 16 presentations of 10 s/75 dB white noise across a 30-min session. During the baseline period prior to the first trial of white noise, we found a main effect of stress (F(1,12) = 36.128, *p* < 0.001), where the stressed group exhibited significantly higher levels of freezing in the stress context relative to the no stress group (Fig. 4a). Additionally, we analyzed freezing behavior across the session during the 10-s pre-noise interval for each trial. While there was no significant change in freezing for time across the session, we saw a significant main effect of stress where the stress group had higher levels of freezing relative to the no stress group (F(1,12) = 79.259, *p* < 0.001), indicating stressed animals remained in post-encounter defense across the session between white-noise trials (Fig. 4b).

Circa-strike, or flight-like/panic-like behavior was measured in 3 ways (Peak Activity Ratio, Frequency of Darting and Velocity). For peak activity ratio (PAR) across the white-noise trials a mixed-factors ANOVA for sex, group, and trial revealed a significant main effect of group (F(1,12) = 102.01, *p* < 0.001) and a main effect of sex (F(1,12) = 7.831, *p* = 0.016). Stressed animals showed significantly higher PAR across the session of white-noise trials compared to the non-stressed group, who showed little change in reactivity to the noise (Fig. 4c). The significant effect of sex shows that regardless of stress condition, males showed a higher PAR than females overall (Online Supplemental Material (OSM) Fig. S1).

For darting analysis, a mixed-factors ANOVA for darts/min across the 16 trials of white noise revealed a significant effect of trial F(15, 180) = 6.742, *p* < 0.001, where all groups showed the greatest dart rate at the start of the session that consistently decreased across the session (Fig. 4d). No other effects were significant for darting behavior.

A close look at velocity data during the circa-strike test revealed robust differences in activity evoked by white noise. Given that darting behavior was highest early in the session (Fig. 4d), we analyzed velocity data averaged across the first four trials of the noise session. A mixed-factors ANOVA revealed a significant time x group interaction (F(75,900) = 11.323, *p* < 0.001) where during the pre-stimulus period, velocity was lower in the stress group compared to the no-stress group, indicative of freezing behavior (pre-stimulus time bins, *p* < 0.05 for stress vs. no stress). Then velocity peaked during the first bins (first 2 s) of the noise period and showed another smaller peak midway through the stimulus then decreased to more stable levels (stress vs. no-stress differences at noise stimulus bins 1–4, 11–13, and 17–18, *p* < 0.05; Fig. 4e). The second peak midway through the noise period in the stress group may reflect a second circastrike-like activity burst in a subset of the animals. We also observed a significant time x sex interaction (F(75,900) = 1.501, *p* = 0.005), where females generally had higher velocity near the middle of the noise stimulus (~4 s) (see OSM Fig. S1B).

When the 16 trials were averaged across the session (Fig. 4f), a mixed-factors ANOVA revealed persistent results with a significant time x group interaction (F(75,900) = 11.8, *p* < 0.001) where the stressed group again showed lower velocity throughout the averaged pre-stimulus period (*p* < 0.05 for bins during pre-stimulus period) and had an initial peak at the beginning and towards the end of the noise stimulus (noise stimulus bins 1, 3–4 (first 2 s), as well as towards the end of the noise stimulus at bins 12, 19, 21 (during ~6 and 9 s; see Fig. 4f). We also found a significant time x sex interaction (F(75, 900) = 2.069, *p* < 0.001), showing a difference in velocity between males and females during the averaged noise period consistent with the first four trials where females had higher velocity around the middle of the noise trials (~3–4 s; see OSM Fig. S1C).

Peak velocity for the stressed group was greater (~15 cm/s) during the early session (first four trials) relative to the average of all 16 trials (~6.3 cm/s), perhaps indicating habituation of this response across trials.

## Discussion

These data show that stress exposure influences defensive behavior across the predatory imminence continuum affecting the modes of pre-encounter, post-encounter, and circa-strike. A significant stressor exposure of ten unsignaled footshocks led to increased anxiety-like behavior in the light gradient open-field task, reduced response to shock and increased freezing following single-shock fear conditioning, and increased panic-like responses to 75 dB white noise when placed back in the stress context in female and male mice.

This study is an extension to our stress-enhanced fear learning (SEFL) model (Rajbhandari et al., 2018; Rau et al., 2005) with an application to the predatory imminence continuum first described in the 1980s (Fanselow & Lester, 1988). According to the PIC, when an animal leaves its nest to forage for food, predatory potential increases and the animal enters the pre-encounter defense mode. Pre-encounter defense is characterized in part by cautious exploration, stretched-approach postures, and reorganized meal patterns by way of increased meal size and reduced time foraging (Fanselow et al., 1988). It is possible that cautious exploration is reflected by the reduced velocity of movement in the open field of stressed mice. Behavioral assays of general exploration in a novel environment are classical tests of anxiety-like behavior, reflecting pre-encounter defense. In the current study we showed that prior stress resulted in reduced velocity in the early phase of novel open-field exploration, suggesting that stress increases the extent of pre-encounter defense when placed in a novel environment.

In the same animals, following single mild-shock contextual fear conditioning, prior stress led to increased duration of freezing behavior, the topographical behavior representing post-encounter defense in rodents. We also observed reduced shock reactivity to the single shock in the stressed group compared to no stress, which may reflect an analgesic response as a result of prior stress in a heightened pre-encounter defense state (Fanselow, 1984b; Lester & Fanselow, 1985). The change from slow velocity exploration to freezing following administration of a footshock reflects the switch in topographical behaviors from pre- to post-encounter defense. In the case of post-encounter defense, the context where the single shock occurred reflects the cue that signals an aversive encounter. This finding is consistent with a recent paper by Hassien et al. demonstrating that a prior footshock stressor supports both associative and non-associative fear (Hassien et al., 2020) as well as our SEFL model of fear sensitization (Perusini et al., 2016; Poulos et al., 2015; Rajbhandari et al., 2018).

Finally, the same animals were returned to the initial stress context and exposed to several brief presentations of 75 dB white noise. During the baseline period prior to the first noise exposure, not surprisingly the stressed group displayed robust freezing behavior compared to little freezing in the no-stress group, indicative of post-encounter defense state, i.e., associative contextual fear from the initial stressor experience. When exposed to trials of novel white noise, freezing animals in the stress group displayed vigorous bursts of activity including running, jumping, or “darting” behavior, indicative of rapid defense mode switching from post-encounter to circa-strike behavior. This phenotype was measured in several ways. We developed an index called the peak activity ratio (PAR), which reflects the sudden change in motion from just prior to the stimulus onset to maximum motion during the noise presentation for each trial (Fanselow et al., 2019). We found that while the no-stress group showed a PAR that hovered around 0.5 for the duration of the session, indicating no change from baseline activity, the stressed group showed robust elevation in PAR throughout the duration of the session, indicating heightened reactivity to 75 dB white noise in the stress context. However, the different levels of pre-stimulus activity between the two stress conditions likely reflect the different amounts of freezing between the groups, which could possibly influence the detected differences in PAR. We also measured the frequency of darting behavior, a measure adapted from Gruene et al. (2015). This measure applied a velocity threshold based on multiple series of experiments in our lab under similar conditions in mice (Trott et al., submitted). Under this definition, we did not observe differences in the frequency of darting behavior between stress and no-stress conditions. The finding that stress had a greater impact on PAR than Darts suggests that stress impacted the magnitude more than the frequency of activity bursts.

Additional support for the hypothesis that stress affected the magnitude of the noise-evoked activity burst comes from the microanalysis of velocity across white noise trials. We analyzed velocity across the pre-stimulus, noise, and post-stimulus period across each trial in the circa-strike noise test in bins of 0.533 s. This analysis revealed that while stressed animals showed reduced velocity during the pre-stimulus period across trials, they displayed robust and peak velocity at the onset of the noise stimulus (within the first second) that was greatest at the beginning of the session (first four trials, Fig. 4e). These measures emphasize the transition in defense-mode switching between post-encounter to circa-strike upon a sudden stimulus change, that does not have to be conditioned, or a painful stimulus (Fadok et al., 2017; Fanselow, 1984a; Hersman et al., 2020). Regardless of the influence that differences in freezing across trials had on PAR effects, the velocity analysis adds a clear picture of noise-evoked change in activity early (Fig. 4e) and across the whole session (Fig. 4f), because unlike PAR it is an absolute measure of the response and is not taken relative to baseline, as is PAR. Taken together, the current findings demonstrate a novel approach to assessing how prior stress leads to a consistent shift in defense mode transition from pre-encounter, to post-encounter, to circa-strike in quantifiable behaviors.

This study used both female and male mice, and did not find any significant sex × stress interactions. This supports the generalizability of the effects of prior stress on PIC defense state shifts across the sexes. However, we do acknowledge the small group sizes accounting for sex (*n* = 4/sex/condition) that may limit the detection of sex effects and interactions, which will be a focus of future evaluation in our model. Still, we did observe some main effects of sex that were not dependent on stress history. In the light gradient open-field task for pre-encounter (anxiety-like behavior), and during the noise stimulus period during the white noise test for circa-strike, we saw increased velocity a few seconds following the peak velocity burst in females relative to males across averaged trials (yet males showed higher overall PARs). The pre-encounter finding is not surprising given that, in general, adult female rodents tend to ambulate more than males (Archer, 1975; Valle & Bols, 1976). One study on conditional fear behavior suggested that females display increased rates of CS-elicited darting behavior (Gruene et al., 2015), which may reflect a lowered threshold to transition from post-encounter to circa-strike when an animal is in a heightened fearful state. However, non-associative effects were not addressed in that study. Nonetheless, reports from our lab and others ( Fanselow et al., 2019; Totty et al., 2021; Trott et al., submitted) found no such sex differences on these circa-strike-related behaviors. In fact, in the current study we observed that male mice had an overall higher PAR than females (Day 5), regardless of stress history. Other studies from our lab in both rats and mice echo similarities between sexes across these behavior systems and support the generalizability of significant stress causing shifts across defense modes.

An important aspect of behavior systems is that prior learning has a profound impact on subsequent responses to species-typical cues, such as in the Pavlovian modification of sexual behavior (Domjan & Gutierrez, 2019). Using the behavior systems framework, the substrate for learning involves an integrated complex of behavior modes to achieve a significant biological function (Domjan & Gutierrez, 2019). In both appetitive and defense systems, the ultimate biological function is evolutionarily adaptive in survival and species maintenance. In contrast to Timberlake’s view on the structure of behavior systems (Timberlake, 1994), Domjan’s approach on sexual behavior centers on characterizing learning systems (Domjan & Gutierrez, 2019). Prior to a learning event, the organization of unconditional behavior systems are organized in a hierarchical system of behavior modes. For both food and mate seeking, these modes are organized as general search, focal search, and consummatory behavior, where the organism’s goal is moving toward terminal modes. The defense system is the opposite, organized as described in the PIC, where antipredator behavior is directed at moving away from terminal modes (predator contact in circa-strike). In both systems prior learning influences response modes. Domjan’s body of work illustrates how Pavlovian conditioning shapes the sexual behavior system as a result of prior sexual experience (Akins et al., 1994; Burns & Domjan, 2001; Domjan, 1994; Domjan & Gutierrez, 2019). The current study supports this stance in the defense behavior system where prior experience with stress creates an adaptation in response across defense modes that is context-dependent as demonstrated in the circa-strike test, which was conducted in the major stress context. This outcome has also been shown in fish with prior predation experience in increasing survival rates and fast-start swimming behavior (Fu et al., 2019), which may reflect a circa-strike SSDR. The behavior systems framework provides a platform to study and organize how organisms acquire evolutionary adaptation and promote survival.

While activation of the defense system is useful for survival in an acute life-threatening setting, there is notable evidence that prior significant stress can come at a cost and is the basis of stress and trauma-related disorders. Decades of research on the behavioral and neurobiological effects of significant stress have shared the ongoing collective goal to better understand the underlying consequences of trauma-related disorders (Battaglia & Ogliari, 2005; Cohen et al., 2013; Goswami et al., 2013; Lister, 1990). Thus, while we are not the first to show that significant acute stress affects anxiety-like, fear, and panic-like behaviors in an animal model, this is the first study to demonstrate how stress causes a consistent shift across modes of defense within subjects. Our approach, which addresses changes across the topographically organized modes in the defense behavior system, allows a comprehensive picture on how a primary manipulation affects relative states of anxiety-like, fear, and panic-like behaviors relevant to clinical mental health conditions. While the behavior systems approach was developed, primarily, to understand the rules by which animals adopt specific behavioral topographies, it may also be useful in advancing understanding of clinical states. For example, Bouton et al. (2001) applied the predatory imminence continuum to anxiety disorders and suggested that post-encounter states may potentiate panic reactions. Here we saw that panic responses only occurred in animals that were in a context that had a strong association with shock.

The predatory imminence continuum defines the defensive behavior system across organized topographical behaviors. The current study integrates features and concepts from decades of research on behavior systems and learning theory and proposes a novel and efficient approach to study the behavioral and neurobiological consequences of significant stress. Empirically, we showed consistent shifts in defense-mode switching in a behavior protocol designed to assess changes in defense response strategy across the PIC. Importantly, this system is highly conserved across species and can provide a framework for studying human mental health conditions that affect defensive states such as anxiety, fear, and panic (Bouton et al., 2001; Mobbs et al., 2009; Perusini & Fanselow, 2015). This research could therefore work to reveal how such responses become maladaptive in human clinical populations following traumatic stress, potentially leading to a shift in defense state toward higher levels of predatory imminence and greater defensive intensity.

## Supplementary Material

Supp

## References

[R1] Akins CK, Domjan M, & Gutierrez G (1994). Topography of sexually conditioned behavior in male Japanese quail (Coturnix japonica) depends on the CS-US interval. Journal of Experimental Psychology. Animal Behavior Processes, 20(2), 199–209. Retrieved from https://www.ncbi.nlm.nih.gov/pubmed/8189188 8189188

[R2] American, Psychiatric, & Association. (2013). Diagnostic and statistical manual of mental disorders (5th ed.). American Psychiatric Publishing.

[R3] Archer J (1975). Rodent sex differences in emotional and related behavior. Behavioral Biology, 14(4), 451–479. 10.1016/s0091-6773(75)90636-7 1100044

[R4] Battaglia M, & Ogliari A (2005). Anxiety and panic: from human studies to animal research and back. Neuroscience and Biobehavioral Reviews, 29(1), 169–179. 10.1016/j.neubiorev.2004.06.013 15652264

[R5] Bolles RC (1970). Species-specific defense reactions and avoidance learning. Psychological Review 77(1), 32–48.

[R6] Bouton ME, Mineka S, & Barlow DH (2001). A modern learning theory perspective on the etiology of panic disorder. Psychological Review, 108(1), 4–32. Retrieved from https://www.ncbi.nlm.nih.gov/pubmed/11212632 11212632 10.1037/0033-295x.108.1.4

[R7] Burns M, & Domjan M (2001). Topography of spatially directed conditioned responding: effects of context and trial duration. Journal of Experimental Psychology. Animal Behavior Processes, 27(3), 269–278. Retrieved from https://www.ncbi.nlm.nih.gov/pubmed/11497325 11497325

[R8] Cohen H, Matar MA, & Joseph Z (2013). Animal models of post-traumatic stress disorder. Current Protocols in Neuroscience, Chapter 9, Unit 9 45. 10.1002/0471142301.ns0945s64 23853112

[R9] Domjan M (1994). Formulation of a behavior system for sexual conditioning. Psychonomic Bulletin & Review, 1(4), 421–428. 10.3758/BF03210946 24203550

[R10] Domjan M, & Gutierrez G (2019). The behavior system for sexual learning. Behavioral Processes, 162, 184–196. 10.1016/j.beproc.2019.01.013 30831223

[R11] Fadok JP, Krabbe S, Markovic M, Courtin J, Xu C, Massi L, … Luthi A (2017). A competitive inhibitory circuit for selection of active and passive fear responses. Nature, 542(7639), 96–100. 10.1038/nature21047 28117439

[R12] Fanselow MS (1984a). Opiate modulation of the active and inactive components of the postshock reaction: parallels between naloxone pretreatment and shock intensity. Behavioral Neuroscience, 98(2), 269–277. Retrieved from https://www.ncbi.nlm.nih.gov/pubmed/6721925 6721925 10.1037//0735-7044.98.2.269

[R13] Fanselow MS (1984b). Shock-induced analgesia on the formalin test: effects of shock severity, naloxone, hypophysectomy, and associative variables. Behavioral Neuroscience, 98(1), 79–95. Retrieved from https://www.ncbi.nlm.nih.gov/pubmed/6320845 6320845 10.1037//0735-7044.98.1.79

[R14] Fanselow MS, & Lester LS (1988). A functional behavioristic approach to aversively motivated behavior: Predatory imminence as a determinant of the topography of defensive behavior. In Bolles RC & Beecher MD (Eds.), Evolution and Learning (pp. 185–211). Erlbaum.

[R15] Fanselow MS, Lester LS, & Helmstetter FJ (1988). Changes in feeding and foraging patterns as an antipredator defensive strategy: a laboratory simulation using aversive stimulation in a closed economy. Journal of the Experimental Analysis of Behavior, 50(3), 361–374. 10.1901/jeab.1988.50-361 3209954 PMC1338904

[R16] Fanselow MS, Hoffman AN, & Zhuravka I (2019). Timing and the transition between modes in the defensive behavior system. Behavioural Processes, 166, 103890. 10.1016/j.beproc.2019.103890 31254627 PMC7108871

[R17] Fu C, Cao ZD, & Fu SJ (2019). Predation experience underlies the relationship between locomotion capability and survival. Comparative Biochemistry and Physiology, Part A, 227, 32–38. 10.1016/j.cbpa.2018.09.005 30236912

[R18] Godsil BP, & Fanselow MS (2004). Light stimulus change evokes an activity response in the rat. Learning & Behavior, 32(3), 299–10. 10.3758/bf03196029 15672825

[R19] Godsil BP, Blackmore MA, & Fanselow MS (2005). Modulation of an activity response with associative and nonassociative fear in the rat: a lighting differential influences the form of defensive behavior evoked after fear conditioning. Learning & Behavior, 33(4), 454–463. 10.3758/bf03193184 16573216

[R20] Goswami S, Rodriguez-Sierra O, Cascardi M, & Pare D (2013). Animal models of post-traumatic stress disorder: face validity. Frontiers in Neuroscience, 7, 89. 10.3389/fnins.2013.00089 23754973 PMC3668155

[R21] Gruene TM, Flick K, Stefano A, Shea SD, & Shansky RM (2015). Sexually divergent expression of active and passive conditioned fear responses in rats. Elife, 4, e11352. 10.7554/eLife.11352 26568307 PMC4709260

[R22] Hassien AM, Shue F, Bernier BE, & Drew MR (2020). A mouse model of stress-enhanced fear learning demonstrates extinction-sensitive and extinction-resistant effects of footshock stress. Behavioural Brain Research, 379, 112391. 10.1016/j.bbr.2019.112391 31785363 PMC7006373

[R23] Hersman S, Allen D, Hashimoto M, Brito SI, & Anthony TE (2020). Stimulus salience determines defensive behaviors elicited by aversively conditioned serial compound auditory stimuli. Elife, 9, e53803. 10.7554/eLife.53803 32216876 PMC7190350

[R24] Lester LS, & Fanselow MS (1985). Exposure to a cat produces opioid analgesia in rats. Behavioral Neuroscience, 99(4), 756–759. Retrieved from https://www.ncbi.nlm.nih.gov/pubmed/3843739 3843739 10.1037//0735-7044.99.4.756

[R25] Lister RG (1990). Ethologically-based animal models of anxiety disorders. Pharmacology & Therapeutics, 46(3), 321–340. 10.1016/0163-7258(90)90021-s 2188266

[R26] Meyer EM, Long V, Fanselow MS, & Spigelman I (2013). Stress increases voluntary alcohol intake, but does not alter established drinking habits in a rat model of posttraumatic stress disorder. Alcoholism: Clinical and Experimental Research, 37(4), 566–574. 10.1111/acer.12012 23126586 PMC3567303

[R27] Mobbs D, Marchant JL, Hassabis D, Seymour B, Tan G, Gray M, … Frith CD (2009). From threat to fear: the neural organization of defensive fear systems in humans. Journal of Neuroscience, 29(39), 12236–12243. 10.1523/JNEUROSCI.2378-09.2009 19793982 PMC2782300

[R28] Perusini JN, & Fanselow MS (2015). Neurobehavioral perspectives on the distinction between fear and anxiety. Learning and Memory, 22(9), 417–425. 10.1101/lm.039180.115 26286652 PMC4561408

[R29] Perusini JN, Meyer EM, Long VA, Rau V, Nocera N, Avershal J, … Fanselow MS (2016). Induction and Expression of Fear Sensitization Caused by Acute Traumatic Stress. Neuropsy-chopharmacology, 41(1), 45–57. 10.1038/npp.2015.224 PMC4677128 26329286

[R30] Poulos AM, Zhuravka I, Long V, Gannam C, & Fanselow M (2015). Sensitization of fear learning to mild unconditional stimuli in male and female rats. Behavioral Neuroscience, 129(1), 62–67. 10.1037/bne0000033 25621793

[R31] Rajbhandari AK, Gonzalez ST, & Fanselow MS (2018). Stress-enhanced fear learning, a robust rodent model of post-traumatic stress disorder. Journal of Visualized Experiments (JOVE), 140, 58306. 10.3791/58306 PMC6235522 30371665

[R32] Rau V, DeCola JP, & Fanselow MS (2005). Stress-induced enhancement of fear learning: an animal model of posttraumatic stress disorder. Neuroscience and Biobehavioral Reviews, 29(8), 1207–1223. 10.1016/j.neubiorev.2005.04.010 16095698

[R33] Timberlake W (1994). Behavior systems, associationism, and Pavlovian conditioning. Psychonomic Bulletin & Review, 1(4), 405–420. 10.3758/BF03210945 24203549

[R34] Totty MS, Warren N, Huddleston I, Ramanathan KR, Ressler RL, Oleksiak CR, & Maren S (2021). Behavioral and brain mechanisms mediating conditioned flight behavior in rats. Scientific Reports, 11(1), 8215. 10.1038/s41598-021-87559-3 33859260 PMC8050069

[R35] Valle FP, & Bols RJ (1976). Age factors in sex differences in open-field activity of rats. Animal Learning & Behavior, 4(4), 457–460. 10.3758/BF03214439

