# Supplementary material for: Anxiety, fear, panic: An approach to assessing the defensive behavior system across the predatory imminence continuum
Source: Learn Behav. Author manuscript; Available in PMC 2023 Sep 1. (PMC9343476; doi:10.3758/s13420-021-00509-x)
Supplement: Supplementary file 1 [file NIHMS1790372-supplement-Supp.pdf]

Supplemental Figure

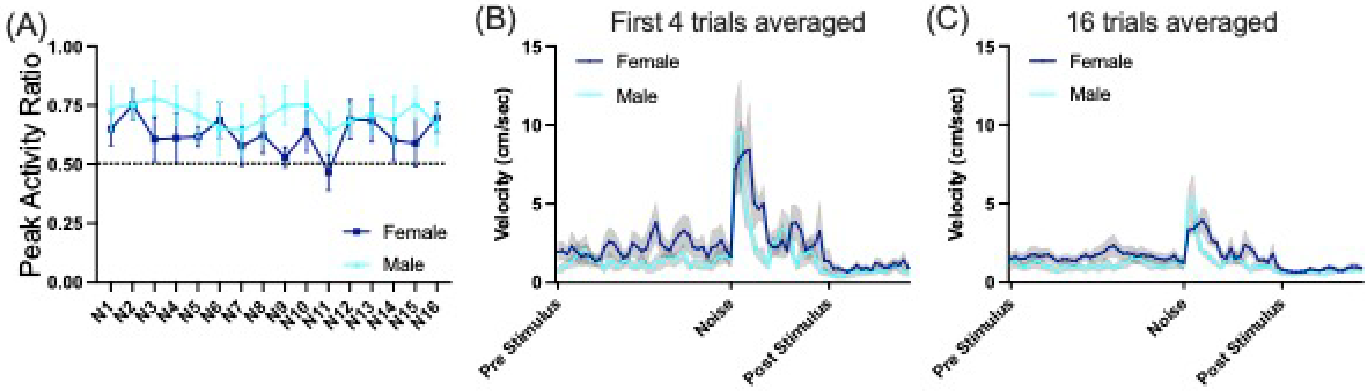

Figure S1. General sex differences in circa-strike test (reactivity to white noise). After finding significant effects of sex in our initial 2way ANOVA, we broke down these observed effects for the circa-strike test. (A) Regardless of stress history, males displayed overall higher peak activity ratio (PAR) across the session compared to females (main effect of sex, $p<0.05$). (B-C) A significant time x sex interaction across micro bins (0.533sec) during session showed that regardless of stress history, females overall had higher velocity during the middle of the averaged white noise trials (B; first 4 trials averaged females higher ~4sec; C: total session averaged females higher ~3-4sec). Together, these analyses show that males exhibited a greater initial peak reaction to the noise, whereas females showed sustained reactivity across the averaged 10sec noise period. n=8/sex with stress conditions collapsed.
